# Supplementary material for: A multi-omics analysis identifies molecular features associated with fertility in heifers (Bos taurus)
Source: Sci Rep. 2023 Aug 4;13:12664. doi: 10.1038/s41598-023-39858-0 (PMC10403585; doi:10.1038/s41598-023-39858-0)
Supplement: Supplementary file 1 — Supplementary Information 1. [file 41598_2023_39858_MOESM1_ESM.html]

Supplementary code to A multi-omics analysis identifies molecular features associated with fertility in heifers (Bos taurus)


# Supplementary code to A multi-omics analysis identifies molecular features associated with fertility in heifers (Bos taurus)

#### Mackenzie Marrella and Fernando H. Biase

#### 2023-07-03


# Overview

Code produced by Fernando Biase, and Mackenzie Marrella. We created
this file to permit reproducibility of the findings described in the
paper. Please direct questions to Fernando Biase:
***fbiase*** at ***vt.edu***

The raw transcriptome data is deposited on GEO repository under the
following access GSE220220.
The normalized protein data obtained from the facility is deposited in
the ProteomeXchange repository under the following access PXD038756.

Please contact Fernando Biase: ***fbiase*** at
***vt.edu*** if you’d like access to other files
used in this code. Updated information may be obtained at www.biaselaboatory.com

**ABSTRACT** **Background**: Infertility or
subfertility is a critical barrier to sustainable cattle production,
including in heifers. The development of heifers that do not produce a
calf within an optimum window of time is a critical factor for the
profitability and sustainability of the cattle industry. In parallel,
heifers are an excellent biomedical model for understanding the
underlying etiology of infertility because well-nourished heifers can
still be infertile, mostly because of inherent physiological and genetic
causes.

**Methods**: Using a high-density single nucleotide
polymorphism (SNP) chip, we collected genotypic data, which were
analyzed using an association analysis in PLINK with Fisher’s exact
test. We also produced quantitative transcriptome data and proteome
data. Transcriptome data were analyzed using the quasi-likelihood test,
Wald’s test, and the likelihood test and proteome data were analyzed
using a generalized mixed model and Student’s t-test.

**Results**: We identified two SNPs significantly
associated with heifer fertility (rs110918927, chr12: 85648422, P =
6.7x10^-7^; and rs109366560, chr11:37666527, P = 2.6x10^-5^). We identified
two genes with differential transcript abundance (eFDR ≤ 0.002) between
the two groups (Fertile and Sub-Fertile): Adipocyte Plasma Membrane
Associated Protein (APMAP, 1.16 greater abundance in the Fertile group)
and Dynein Axonemal Intermediate Chain 7 (DNAI7, 1.23 greater abundance
in the Sub-Fertile group). Our analysis revealed that the protein
Alpha-ketoglutarate-dependent dioxygenase FTO was more abundant in the
plasma collected from Fertile heifers relative to their Sub-Fertile
counterparts (FDR < 0.05). Lastly, an integrative analysis of the
three datasets identified a series of molecular features (SNPs, gene
transcripts, and proteins) that discriminated 21 out of 22 heifers
correctly based on their fertility category.

**Conclusions**: Our multi-omics analyses confirm the
complex nature of female fertility. Very importantly, our results also
highlight differences in the molecular profile of heifers associated
with fertility that transcend the constraints of breed-specific genetic
background.

---

```
library('dplyr', quietly = TRUE,lib.loc="/usr/lib/R/site-library")
library('ggplot2', quietly = TRUE,lib.loc="/usr/lib/R/site-library")
library('edgeR',quietly = TRUE, lib.loc="/usr/lib/R/site-library")
library("cowplot", quietly = TRUE,lib.loc="/usr/lib/R/site-library")
library("GGally",quietly = TRUE,lib.loc="/usr/lib/R/site-library")
library("DESeq2",quietly = TRUE,lib.loc="/usr/lib/R/site-library")
library('readxl',quietly = TRUE,lib.loc="/usr/lib/R/site-library")
library("VennDiagram", quietly = TRUE, lib.loc="/usr/lib/R/site-library")
library("ComplexHeatmap",quietly = TRUE, lib.loc="/usr/lib/R/site-library")
library("flashClust", quietly = TRUE, lib.loc="/usr/lib/R/site-library")
library('plotly' , quietly = TRUE, lib.loc="/usr/lib/R/site-library")
library('tidyverse' ,quietly = TRUE , lib.loc="/usr/lib/R/site-library")
library('htmlwidgets' , quietly = TRUE, lib.loc="/usr/lib/R/site-library")
library('reshape2' ,quietly = TRUE, lib.loc="/usr/lib/R/site-library")
library("ggpubr" ,quietly = TRUE, lib.loc="/usr/lib/R/site-library")
library("car" ,quietly = TRUE, lib.loc="/usr/lib/R/site-library") 
library("goseq" ,quietly = TRUE, lib.loc="/usr/lib/R/site-library")
library("stringr",quietly = TRUE , lib.loc="/usr/lib/R/site-library")
library("data.table",quietly = TRUE , lib.loc="/usr/lib/R/site-library")
library("tidyr",quietly = TRUE , lib.loc="/usr/lib/R/site-library")
library("dplyr",quietly = TRUE , lib.loc="/usr/lib/R/site-library")
library("ggsignif",quietly = TRUE , lib.loc="/usr/lib/R/site-library")
library("kableExtra",quietly = TRUE , lib.loc="/usr/lib/R/site-library")
library("grid",quietly = TRUE , lib.loc="/usr/lib/R/site-library")
library("gridExtra",quietly = TRUE , lib.loc="/usr/lib/R/site-library")
library("bigmemory",quietly = TRUE , lib.loc="/usr/lib/R/site-library")
library("doParallel",quietly = TRUE , lib.loc="/usr/lib/R/site-library")
library("ggmanh",quietly = TRUE , lib.loc="/usr/lib/R/site-library")
library("lme4",quietly = TRUE , lib.loc="/usr/lib/R/site-library")
library("emmeans",quietly = TRUE , lib.loc="/usr/lib/R/site-library")
library("limma",quietly = TRUE , lib.loc="/usr/lib/R/site-library") 
library("MultiAssayExperiment",quietly = TRUE , lib.loc="/usr/lib/R/site-library")
library('MOFA2', lib.loc="/usr/lib/R/site-library")
```

# Procedures

Use the tabs below to access each section of our code.

## Get resources for reproducibility

### Obtain Ensembl Annotation

```
library('biomaRt', lib.loc="/usr/lib/R/site-library")
cow<-useMart("ensembl", dataset = "btaurus_gene_ensembl", host="www.ensembl.org") 
annotation.ensembl.symbol<-getBM(attributes = c('ensembl_gene_id','external_gene_name','description','hgnc_symbol','gene_biotype','transcript_length','chromosome_name','start_position','end_position','strand'),  values = "*", mart = cow)

cow<-useMart("ensembl", dataset = "btaurus_gene_ensembl", host="www.ensembl.org") 
annotation.ensembl.transcript<-getBM(attributes = c('ensembl_gene_id','gene_biotype','ensembl_transcript_id','transcript_length'),  values = "*", mart = cow)
annotation.ensembl.transcript<-annotation.ensembl.transcript[annotation.ensembl.transcript$gene_biotype =="protein_coding",]
annotation.ensembl.transcript<-annotation.ensembl.transcript[order(annotation.ensembl.transcript$ensembl_gene_id, -annotation.ensembl.transcript$transcript_length),]
annotation.ensembl.transcript<-annotation.ensembl.transcript[!duplicated(annotation.ensembl.transcript$ensembl_gene_id),]
annotation.ensembl.transcript<-annotation.ensembl.transcript[annotation.ensembl.transcript$transcript_length > 400,]

annotation.ensembl.symbol<-annotation.ensembl.symbol[order(annotation.ensembl.symbol$ensembl_gene_id, -annotation.ensembl.symbol$transcript_length),]
annotation.ensembl.symbol<-annotation.ensembl.symbol[!duplicated(annotation.ensembl.symbol$ensembl_gene_id),]
gene.length<-annotation.ensembl.symbol[,c( "ensembl_gene_id", "transcript_length" )]
annotation.GO.biomart<-getBM(attributes = c('ensembl_gene_id', 'external_gene_name','go_id','name_1006','namespace_1003'),  values = "*", mart = cow)

#write.table(annotation.ensembl.symbol,file="/mnt/storage/lab_folder/shared_R_codes/fernando/angus_holstein_association/resources/2022_05_25_annotation.ensembl.symbol.txt", sep = "\t",append = FALSE, quote = FALSE)
#system('bzip2 --best /mnt/storage/lab_folder/shared_R_codes/fernando/angus_holstein_association/resources/2022_05_25_annotation.ensembl.symbol.txt')

#write.table(gene.length,file="/mnt/storage/lab_folder/shared_R_codes/fernando/angus_holstein_association/resources/2022_05_25_gene.length.txt", sep = "\t",append = FALSE, quote = FALSE)
#system('bzip2 --best /mnt/storage/lab_folder/shared_R_codes/fernando/angus_holstein_association/resources/2022_05_25_gene.length.txt')

#write.table(annotation.GO.biomart,file="/mnt/storage/lab_folder/shared_R_codes/fernando/angus_holstein_association/resources/2022_05_25_annotation.GO.biomart.txt", sep = "\t",append = FALSE, quote = FALSE)
#system('bzip2 --best /mnt/storage/lab_folder/shared_R_codes/fernando/angus_holstein_association/resources/2022_05_25_annotation.GO.biomart.txt')

#write.table(annotation.ensembl.transcript,file="/mnt/storage/lab_folder/shared_R_codes/fernando/angus_holstein_association/resources/2022_05_25_annotation.ensembl.transcript.txt", sep = "\t",append = FALSE, quote = FALSE)
#system('bzip2 --best /mnt/storage/lab_folder/shared_R_codes/fernando/angus_holstein_association/resources/2022_05_25_annotation.ensembl.transcript.txt')
```

### Import Annotation

```
annotation.ensembl.symbol<-read.delim("/mnt/storage/lab_folder/shared_R_codes/fernando/angus_holstein_association/resources/2022_05_25_annotation.ensembl.symbol.txt.bz2", header=TRUE, sep= "\t",row.names=1, stringsAsFactors = FALSE)
gene.length<-read.delim("/mnt/storage/lab_folder/shared_R_codes/fernando/angus_holstein_association/resources/2022_05_25_gene.length.txt.bz2", header=TRUE, sep= "\t",row.names=1, stringsAsFactors = FALSE)
annotation.GO.biomart<-read.delim("/mnt/storage/lab_folder/shared_R_codes/fernando/angus_holstein_association/resources/2022_05_25_annotation.GO.biomart.txt.bz2", header=TRUE, sep= "\t",row.names=1, stringsAsFactors = FALSE)
annotation.ensembl.transcript<-read.delim("/mnt/storage/lab_folder/shared_R_codes/fernando/angus_holstein_association/resources/2022_05_25_annotation.ensembl.transcript.txt.bz2", header=TRUE, sep= "\t",row.names=1, stringsAsFactors = FALSE)
```

## GWAS analysis

### Import raw data and prepare files for Plink

```
#set up phenotype data for analysis
snp_map_genotypes <- read.table("/mnt/storage/lab_folder/heifer_infertility/AI_angus_holstein/assoc_analysis/SNP_Map.txt", header = TRUE, sep = "\t")
ah_genotypes<- data.table::fread("/mnt/storage/lab_folder/heifer_infertility/AI_angus_holstein/assoc_analysis/Virginia_Tech_Univ_Biase_BOV770V01_20220825_FinalReport.txt", header = TRUE, sep = "\t", skip = 9)

ah_genotypes$Sample.ID <- gsub(" ","_",ah_genotypes$Sample.ID)
ah_genotypes <- ah_genotypes[,c(2,2,1,3,4)]
ah_genotypes$Allele1...Forward <- gsub("-","0",ah_genotypes$Allele1...Forward)
ah_genotypes$Allele2...Forward <- gsub("-","0",ah_genotypes$Allele2...Forward)
ah_genotypes<- ah_genotypes[!ah_genotypes$Sample.ID=="Sample_23" & !ah_genotypes$Sample.ID=="Sample_24",]


snp_map_genotypes <- snp_map_genotypes[,c(3,2,4)]


sample_id <- c("Sample_1","Sample_2","Sample_3","Sample_4","Sample_5","Sample_6","Sample_7","Sample_8","Sample_9","Sample_10","Sample_11","Sample_12","Sample_13","Sample_14","Sample_15","Sample_16","Sample_17","Sample_18","Sample_19","Sample_20","Sample_21","Sample_22") 
phenotype_angus_holstein <- c("2","2","2","2","2","1","1","1","1","1","2","2","2","2","2", "2","2","1","1","1","1","1")
missing_row <- c(0,0,0,0,0,0,0,0,0,0,0,0,0,0,0,0,0,0,0,0,0,0)
sex <- c(2,2,2,2,2,2,2,2,2,2,2,2,2,2,2,2,2,2,2,2,2,2)
fam_file <- data.frame(sample_id,sample_id,missing_row,missing_row,sex,phenotype_angus_holstein)


#write.table(ah_genotypes, "/mnt/storage/lab_folder/heifer_infertility/AI_angus_holstein/assoc_analysis/angus_holstein_genotypes.lgen", col.names = FALSE, row.names = FALSE, sep = "\t" , quote = FALSE)
#write.table(snp_map_genotypes, "/mnt/storage/lab_folder/heifer_infertility/AI_angus_holstein/assoc_analysis/angus_holstein_genotypes.map", col.names = FALSE, row.names = FALSE, sep = "\t" , quote = FALSE)
#write.table(fam_file, "/mnt/storage/lab_folder/heifer_infertility/AI_angus_holstein/assoc_analysis/angus_holstein_genotypes.fam", col.names = FALSE, row.names = FALSE, sep = "\t" , quote = FALSE)
```

### Quality control and analysis in Plink

```
system("/home/fbiase/bioinfo/plink --lfile /mnt/storage/lab_folder/heifer_infertility/AI_angus_holstein/assoc_analysis/angus_holstein_genotypes --cow  --recode --out /mnt/storage/lab_folder/heifer_infertility/AI_angus_holstein/assoc_analysis/angus_holstein_genotypes_data")

system("/home/fbiase/bioinfo/plink --file /mnt/storage/lab_folder/heifer_infertility/AI_angus_holstein/assoc_analysis/angus_holstein_genotypes_data --cow --make-bed --out /mnt/storage/lab_folder/heifer_infertility/AI_angus_holstein/assoc_analysis/angus_holstein_genotypes_data")

system("/home/fbiase/bioinfo/plink --bfile /mnt/storage/lab_folder/heifer_infertility/AI_angus_holstein/assoc_analysis/angus_holstein_genotypes_data --cow --test-missing --out /mnt/storage/lab_folder/heifer_infertility/AI_angus_holstein/assoc_analysis/angus_holstein_genotypes_data")

system("perl /mnt/storage/lab_folder/heifer_infertility/AI_angus_holstein/assoc_analysis/run-diffmiss-qc.pl  /mnt/storage/lab_folder/heifer_infertility/AI_angus_holstein/assoc_analysis/angus_holstein_genotypes_data > /mnt/storage/lab_folder/heifer_infertility/AI_angus_holstein/assoc_analysis/fail-diffmiss-qc.txt")

system("/home/fbiase/bioinfo/plink --file /mnt/storage/lab_folder/heifer_infertility/AI_angus_holstein/assoc_analysis/angus_holstein_genotypes_data --cow --mind 0.1   --maf 0.01 --geno 0.05 --hwe 0.00001 --make-bed --autosome --out /mnt/storage/lab_folder/heifer_infertility/AI_angus_holstein/assoc_analysis/angus_holstein_genotypes_qc_data")
#606553 variants and 22 cattle pass filters and QC

system("/home/fbiase/bioinfo/plink --bfile /mnt/storage/lab_folder/heifer_infertility/AI_angus_holstein/assoc_analysis/angus_holstein_genotypes_qc_data --cow --recode --tab --out /mnt/storage/lab_folder/heifer_infertility/AI_angus_holstein/assoc_analysis/angus_holstein_genotypes_qc_data")

system("/home/fbiase/bioinfo/plink --bfile /mnt/storage/lab_folder/heifer_infertility/AI_angus_holstein/assoc_analysis/angus_holstein_genotypes_qc_data  --cow --indep-pairwise 50 5 0.2 --out /mnt/storage/lab_folder/heifer_infertility/AI_angus_holstein/assoc_analysis/angus_holstein_genotypes_qc_data")

system("/home/fbiase/bioinfo/plink --bfile /mnt/storage/lab_folder/heifer_infertility/AI_angus_holstein/assoc_analysis/angus_holstein_genotypes_qc_data --cow  --exclude /mnt/storage/lab_folder/heifer_infertility/AI_angus_holstein/assoc_analysis/angus_holstein_genotypes_qc_data.prune.in --pca --out /mnt/storage/lab_folder/heifer_infertility/AI_angus_holstein/assoc_analysis/angus_holstein_genotypes_qc_data")

system("/home/fbiase/bioinfo/plink --bfile /mnt/storage/lab_folder/heifer_infertility/AI_angus_holstein/assoc_analysis/angus_holstein_genotypes_qc_data --cow  --cluster --K 2 --out /mnt/storage/lab_folder/heifer_infertility/AI_angus_holstein/assoc_analysis/angus_holstein_genotypes_qc_data_cluster")

system("/home/fbiase/bioinfo/plink --bfile /mnt/storage/lab_folder/heifer_infertility/AI_angus_holstein/assoc_analysis/angus_holstein_genotypes_qc_data --cow  --make-rel triangle gz  --out /mnt/storage/lab_folder/heifer_infertility/AI_angus_holstein/assoc_analysis/angus_holstein_genotypes_qc_data_relationship")

system("/home/fbiase/bioinfo/plink --bfile /mnt/storage/lab_folder/heifer_infertility/AI_angus_holstein/assoc_analysis/angus_holstein_genotypes_qc_data --cow --within /mnt/storage/lab_folder/heifer_infertility/AI_angus_holstein/assoc_analysis/angus_holstein_genotypes_qc_data_cluster.cluster2 --exclude /mnt/storage/lab_folder/heifer_infertility/AI_angus_holstein/assoc_analysis/angus_holstein_genotypes_qc_data.prune.in --assoc fisher  --out /mnt/storage/lab_folder/heifer_infertility/AI_angus_holstein/assoc_analysis/angus_holstein_genotypes_qc_data")

system("/home/fbiase/bioinfo/plink --bfile /mnt/storage/lab_folder/heifer_infertility/AI_angus_holstein/assoc_analysis/angus_holstein_genotypes_qc_data --cow --within /mnt/storage/lab_folder/heifer_infertility/AI_angus_holstein/assoc_analysis/angus_holstein_genotypes_qc_data_cluster.cluster2 --exclude /mnt/storage/lab_folder/heifer_infertility/AI_angus_holstein/assoc_analysis/angus_holstein_genotypes_qc_data.prune.in --assoc fisher --seed 6377474 --perm  --out /mnt/storage/lab_folder/heifer_infertility/AI_angus_holstein/assoc_analysis/angus_holstein_genotypes_qc_data")
```

### load the analysis

```
angus_holstein_genotypes_qc_data.fisher<-data.table::fread("/mnt/storage/lab_folder/heifer_infertility/AI_angus_holstein/assoc_analysis/angus_holstein_genotypes_qc_data.assoc.fisher", data.table=FALSE)

angus_holstein_genotypes_qc_data.fisher.perm<-data.table::fread("/mnt/storage/lab_folder/heifer_infertility/AI_angus_holstein/assoc_analysis/angus_holstein_genotypes_qc_data.assoc.fisher.perm", data.table=FALSE)

angus_holstein_genotypes_qc_data.fisher<-merge(angus_holstein_genotypes_qc_data.fisher, angus_holstein_genotypes_qc_data.fisher.perm, by=c("CHR","SNP"), all=TRUE)

angus_holstein_genotypes_qc_data.fisher<-angus_holstein_genotypes_qc_data.fisher<-angus_holstein_genotypes_qc_data.fisher[order(angus_holstein_genotypes_qc_data.fisher$P),]

head(angus_holstein_genotypes_qc_data.fisher)
```

```
##        CHR                    SNP        BP A1    F_A    F_U A2         P
## 105744  12     BovineHD1200026258  89664275  G 0.7083 0.0000  A 6.753e-07
## 86695   11 Hapmap31720-BTA-126418  37519673  G 0.1250 0.7778  A 2.658e-05
## 105735  12     BovineHD1200026243  89607810  G 0.5000 0.0000  A 1.342e-04
## 361032  27     BovineHD2700000503   1491696  C 0.1667 0.7500  T 1.727e-04
## 473766   5     BovineHD0500034893 119441043  G 0.1667 0.7500  A 1.727e-04
## 473767   5     BovineHD0500034894 119441844  G 0.1667 0.7500  T 1.727e-04
##             OR      EMP1      NP
## 105744      NA 0.0000195 1000000
## 86695  0.04082 0.0001431  286567
## 105735      NA 0.0021390   19168
## 361032 0.06667 0.0002232  183683
## 473766 0.06667 0.0008473   48976
## 473767 0.06667 0.0008473   48976
```

```
#write.table(angus_holstein_genotypes_qc_data.fisher, "/mnt/storage/lab_folder/shared_R_codes/fernando/angus_holstein_association/results/Additional_file_2.txt", col.names = TRUE, row.names = FALSE, sep = "\t" , quote = FALSE)
```

### Genotypes per sample

```
ah_genotypes<- data.table::fread("/mnt/storage/lab_folder/heifer_infertility/AI_angus_holstein/assoc_analysis/Virginia_Tech_Univ_Biase_BOV770V01_20220825_FinalReport.txt", header = TRUE, sep = "\t", skip = 9)
ah_genotypes<-ah_genotypes[,c(1,2,3,4)]
colnames(ah_genotypes)<-c("SNP_Name", "Sample_ID","Allele1", "Allele2")
ah_genotypes<- ah_genotypes[!ah_genotypes$Sample_ID=="Sample_23" & !ah_genotypes$Sample_ID=="Sample_24",]

ah_genotypes<-ah_genotypes[ah_genotypes$SNP_Name %in% angus_holstein_genotypes_qc_data.fisher$SNP,]
ah_genotypes$genotype<-paste(ah_genotypes$Allele1,ah_genotypes$Allele2, sep="")
ah_genotypes<-ah_genotypes[!(ah_genotypes$genotype == "--"),]

ah_genotypes %>% group_by(Sample_ID) %>% summarise(n = n()) %>% print(n = 24)
```

```
## # A tibble: 24 × 2
##    Sample_ID      n
##    <chr>      <int>
##  1 Sample 1  574813
##  2 Sample 10 574778
##  3 Sample 11 574705
##  4 Sample 12 574938
##  5 Sample 13 574757
##  6 Sample 14 574715
##  7 Sample 15 574739
##  8 Sample 16 574945
##  9 Sample 17 574960
## 10 Sample 18 574955
## 11 Sample 19 574816
## 12 Sample 2  574705
## 13 Sample 20 574893
## 14 Sample 21 574915
## 15 Sample 22 574850
## 16 Sample 23 574782
## 17 Sample 24 574817
## 18 Sample 3  574592
## 19 Sample 4  574959
## 20 Sample 5  574882
## 21 Sample 6  574902
## 22 Sample 7  574791
## 23 Sample 8  574849
## 24 Sample 9  574909
```

## Transcriptome analysis

### Read count summary

```
files<-list.files("/mnt/storage/lab_folder/heifer_infertility/AI_angus_holstein/alignment3", recursive=T, pattern="summary.txt", full.names = TRUE)
#length(files)

reads_produced_a<-data.frame()
reads_produced_b<-data.frame()
#length(files)

for (n in 1:22) {
reads_produced<-read.delim(files[n], sep= " ",header=FALSE, stringsAsFactors = FALSE, comment.char= "#")
sample<-substring(files[n], 73,74)
number_of_reads_produced<-as.integer(reads_produced[1,1])

reads_produced_a<-data.frame(sample,number_of_reads_produced)
reads_produced_b<-rbind(reads_produced_b,reads_produced_a)
}

files<-list.files("/mnt/storage/lab_folder/heifer_infertility/AI_angus_holstein/counting3", recursive=T, pattern="count.summary", full.names = TRUE)
#length(files)
counting_summary_a<-data.frame()
counting_summary_b<-data.frame()
#length(files)

for (n in 1:22) {
counting_summary<-read.delim(files[n], sep= "\t",header=TRUE, stringsAsFactors = FALSE, comment.char= "#")
sample<-substring(files[n], 72,73)
number_of_reads_assigned<-counting_summary[1,2]
unassigned_NoFeatures<-counting_summary[12,2]
unassigned_Ambiguity<-counting_summary[14,2]
counting_summary_a<-data.frame(sample,number_of_reads_assigned, unassigned_NoFeatures, unassigned_Ambiguity)
counting_summary_b<-rbind(counting_summary_b,counting_summary_a)
}

counting_summary_b$reads_sequenced<-reads_produced_b$number_of_reads_produced
counting_summary_b$reads_retained<-counting_summary_b$number_of_reads_assigned + counting_summary_b$unassigned_NoFeatures + counting_summary_b$unassigned_Ambiguity
counting_summary_b$reads_discarted<-counting_summary_b$reads_sequenced - counting_summary_b$reads_retained
counting_summary_b$perc_number_of_reads_discarted<-counting_summary_b$reads_discarted/counting_summary_b$reads_sequenced
counting_summary_b$perc_number_of_reads_retained<-counting_summary_b$reads_retained/counting_summary_b$reads_sequenced
counting_summary_b$perc_number_of_reads_assigned<-counting_summary_b$number_of_reads_assigned/counting_summary_b$reads_sequenced
counting_summary_b$perc_unassigned_NoFeatures<-counting_summary_b$unassigned_NoFeatures/counting_summary_b$reads_sequenced
counting_summary_b$perc_unassigned_Ambiguity<-counting_summary_b$unassigned_Ambiguity/counting_summary_b$reads_sequenced

#write.table(counting_summary_b, file="/mnt/storage/lab_folder/shared_R_codes/fernando/angus_holstein_association/resources/counting_summary_b_second_alignment.txt",quote = FALSE, sep = "\t",row.names = FALSE)
```

### Plot read count summary

```
counting_summary_c<-reshape2::melt(counting_summary_b[,c(1,8,10:12)])
```

```
## Using sample as id variables
```

```
counting_summary_c$value<-counting_summary_c$value*100
counting_summary_c$variable<-factor(counting_summary_c$variable, levels=c( "perc_number_of_reads_discarted" ,"perc_unassigned_Ambiguity","perc_unassigned_NoFeatures" , "perc_number_of_reads_assigned"))
plot_a<-ggplot() + 
  geom_bar(aes(y = value, x = sample, fill = variable), data = counting_summary_c,stat="identity")+
  scale_y_continuous(name="Percentage", breaks = seq(0,100,10))+
  scale_fill_hue(labels=c("Discarted", "Unassigned_Ambiguity","Unassigned_NoFeatures" , "Assigned"))+
  #ggtitle("Distribution of reads after alignment")+
  theme_bw(base_size = 12)+
  theme(legend.title = element_blank(),
        axis.title.x = element_blank(),
        axis.text.x = element_text(angle=75, hjust=1))

counting_summary_c<-reshape2::melt(counting_summary_b[,c(1,5)])
```

```
## Using sample as id variables
```

```
plot_b<-ggplot() + 
  geom_bar(aes(y = value, x = sample, fill = variable), data = counting_summary_c,stat="identity")+
  scale_y_continuous(name="Read pairs sequenced")+
  theme_bw(base_size = 12)+
  theme(legend.title = element_blank(),
        axis.title.x = element_blank(),
        axis.text.x = element_blank())

counting_summary_c<-reshape2::melt(counting_summary_b[,c(1,2)])
```

```
## Using sample as id variables
```

```
plot_c<-ggplot() + 
  geom_bar(aes(y = value, x = sample, fill = variable), data = counting_summary_c,stat="identity")+
  scale_y_continuous(name="Read pairs in annotation")+
  geom_hline(yintercept=10^7, linetype="dashed", color = "gray", size=1)+
  theme_bw(base_size = 12)+
  theme(legend.title = element_blank(),
        axis.title.x = element_blank(),
        axis.text.x = element_blank())
```

```
## Warning: Using `size` aesthetic for lines was deprecated in ggplot2 3.4.0.
## ℹ Please use `linewidth` instead.
## This warning is displayed once every 8 hours.
## Call `lifecycle::last_lifecycle_warnings()` to see where this warning was
## generated.
```

```
counting_summary_c<-reshape2::melt(counting_summary_b[,c(1,10)])
```

```
## Using sample as id variables
```

```
plot_d<-ggplot() + 
  geom_bar(aes(y = value, x = sample, fill = variable), data = counting_summary_c,stat="identity")+
  scale_y_continuous(name="Proportion reads \n matching annotation")+
  theme_bw(base_size = 12)+
  theme(legend.title = element_blank(),
        axis.title.x = element_blank(),
        axis.text.x = element_blank())

plot_grid( plot_b, plot_c,plot_d, plot_a,  ncol = 1, align = 'v')
```

### Import Sample Information

```
sample_info <- as.data.frame(read_excel("/mnt/storage/lab_folder/shared_R_codes/fernando/angus_holstein_association/resources/sample_info.xlsx"))
sample_info$sample<- as.factor(sample_info$sample)
sample_info$preg<- as.factor(sample_info$preg)
sample_info$breed<- as.factor(sample_info$breed)
```

### Counts per sample

```
files<-list.files("/mnt/storage/lab_folder/heifer_infertility/AI_angus_holstein/counting3", recursive=T, pattern="count", full.names = TRUE)
files<-files[grep("summary", files, invert = TRUE)]
files<-files[grep(".count1", files, invert = TRUE)]
files<-files[grep(".count2", files, invert = TRUE)]
files<-files[grep(".sh", files, invert = TRUE)]
#length(files)
count_data<-data.frame(matrix(nrow=27607))
for (n in 1:22) {
  count<-read.delim(files[n], header=TRUE, sep= "\t", stringsAsFactors = FALSE, comment.char= "#")
  count<-count[,c(1,7)]
  count_data<-cbind(count_data,count)
}
rownames(count_data)<-count_data[,2]
count_data<-count_data[,seq(from = 3, to = 45, by = 2)]
colnames(count_data)<- substr(colnames(count_data), 74, 75)

colnames(count_data)<-sample_info$Animal_ID
#colnames(count_data)<-sample_info$SNP_array_ID
#count_data_export<-data.frame(gene_id=rownames(count_data),count_data)
#write_delim(count_data_export, file = "/mnt/storage/lab_folder/shared_R_codes/fernando/angus_holstein_association/resources/2022_12_02_unfiltered_count_data.txt", delim = "\t", quote =  "none")
#system("bzip2 /mnt/storage/lab_folder/shared_R_codes/fernando/angus_holstein_association/resources/2022_12_02_unfiltered_count_data.txt")
```

### Sequencing summary

```
count_data_a<-count_data[rowSums(count_data)>0,]
count_data_annotated<-merge(count_data_a,annotation.ensembl.symbol, by.x="row.names", by.y="ensembl_gene_id", all.x=TRUE, all.y=FALSE)

n_reads_protein_coding<-sum(count_data_annotated[count_data_annotated$gene_biotype=='protein_coding',c(2:6)])
n_reads_lncRNA<-sum(count_data_annotated[count_data_annotated$gene_biotype=='lncRNA',c(2:6)])
n_reads_pseudogene<-sum(count_data_annotated[count_data_annotated$gene_biotype %in% c('pseudogene','processed_pseudogene'),c(2:6)])
n_reads_others<-sum(count_data_annotated[!(count_data_annotated$gene_biotype %in% c('pseudogene','processed_pseudogene','protein_coding','lncRNA')),c(2:6)])

summary_RNA_seq<-data.frame(class=c('protein_coding','lncRNA', 'pseudogene','others'), nreads=c(n_reads_protein_coding,n_reads_lncRNA,n_reads_pseudogene,n_reads_others))

summary_RNA_seq<-mutate(summary_RNA_seq,  prop = round(nreads/sum(nreads)*100,2))
summary_RNA_seq<-summary_RNA_seq[with(summary_RNA_seq, order(-nreads)),]
summary_RNA_seq$class<-factor(summary_RNA_seq$class, levels=c('protein_coding', 'lncRNA','pseudogene','others' ))

knitr::kable(summary_RNA_seq, format = 'pandoc')
```

|  | class | nreads | prop |
| --- | --- | --- | --- |
| 1 | protein\_coding | 123653221 | 98.85 |
| 4 | others | 570242 | 0.46 |
| 3 | pseudogene | 442795 | 0.35 |
| 2 | lncRNA | 422486 | 0.34 |

### Subset for protein-coding, lncRNAs, and pseudogenes

```
count_data_a<-count_data[rowSums(count_data)>0,]
count_data_annotated<-merge(count_data_a,annotation.ensembl.symbol, by.x="row.names", by.y="ensembl_gene_id", all.x=TRUE, all.y=FALSE)
count_data_annotated<-count_data_annotated[count_data_annotated$gene_biotype %in% c('protein_coding', 'lncRNA','pseudogene'),]
count_data_annotated_length<-gene.length[gene.length$ensembl_gene_id %in% count_data_annotated$Row.names, 2]
```

### Calculate CPM, FPKM, TPM

```
count_data_b<-count_data_annotated[,c(2:23)]
rownames(count_data_b)<-count_data_annotated$Row.names

lib_size <- base::colSums(count_data_b)
norm_factors <- edgeR::calcNormFactors(object = count_data_b, lib.size = lib_size, method = "TMM")

#FPKM
data_fpkm<-edgeR::rpkm(sweep(count_data_b, 2, norm_factors, "/"), count_data_annotated_length)

#CPM
data_cpm<-edgeR::cpm(sweep(count_data_b, 2, norm_factors, "/"),normalized.lib.sizes = TRUE,log = FALSE)

#TPM
x <- count_data_b / count_data_annotated_length
data_tpm <- data.frame(t( t(x) * 1e6 / colSums(x) ))
rownames(data_tpm)<-count_data_annotated$Row.names

keep<-rowSums(data_fpkm>1) >=5
data_fpkm_filtered<-data_fpkm[keep,]
keep<-rowSums(data_cpm>1) >=5
data_cpm_filtered<-data_cpm[keep,]
genes_expressed<-intersect(rownames(data_fpkm_filtered), rownames(data_cpm_filtered))

data_tpm<-data_tpm[rownames(data_tpm) %in% genes_expressed,]

data_fpkm_filtered<-data_fpkm_filtered[rownames(data_fpkm_filtered) %in% genes_expressed,]
data_cpm_filtered<-data_cpm_filtered[rownames(data_cpm_filtered) %in% genes_expressed,]

count_data_filtered<-count_data[rownames(count_data) %in% genes_expressed,]
```

### Gene summary

```
table(annotation.ensembl.symbol[annotation.ensembl.symbol$ensembl_gene_id %in% genes_expressed, ]$gene_biotype)
```

```
## 
##         lncRNA protein_coding     pseudogene 
##            228          12105            112
```

### DEG Analysis - Fertility

```
heifer_data <- sample_info

#edgeR
preg<-factor(heifer_data$preg, levels=c("P", "NP"))
breed<-factor(heifer_data$breed, levels=c("ANG","HOL"))
heifer_design<- model.matrix(~ heifer_data$breed + heifer_data$preg)

heifer <-edgeR::DGEList(count=count_data_filtered, group=heifer_data$preg)
heifer <-edgeR::estimateDisp(heifer, heifer_design, robust=TRUE)
heifer_QLFit <- edgeR::glmQLFit(heifer, heifer_design, robust=TRUE)
heifer_QLF <- edgeR::glmQLFTest(heifer_QLFit, coef="heifer_data$pregP")
heifer_edgeR_results_QLF<- edgeR::topTags(heifer_QLF, adjust.method = "fdr", n=Inf)$table

#DESeq2 wald
heifer_DESeq <- DESeq2::DESeqDataSetFromMatrix(countData=count_data_filtered, colData=heifer_data, design= ~ breed + preg)
heifer_Wald<-DESeq2::DESeq(heifer_DESeq, test="Wald")
heifer_DESeq_results_Wald<-DESeq2::results(heifer_Wald, contrast=c("preg",  "P", "NP"), pAdjustMethod="fdr", tidy=TRUE)

#DESeq2 LRT
heifer_LRT<-DESeq2::DESeq(heifer_DESeq, test="LRT", full= ~ breed + preg, reduced= ~ breed )
heifer_DESeq_results_LRT<-DESeq2::results(heifer_LRT, contrast=c("preg",  "P", "NP"), pAdjustMethod="fdr", tidy=TRUE)


heifer_edgeR_results_QLF_heifer_DESeq_results_Wald_LRT<-merge(heifer_edgeR_results_QLF,heifer_DESeq_results_Wald, by.x="row.names", by.y="row")
heifer_edgeR_results_QLF_heifer_DESeq_results_Wald_LRT<-merge(heifer_edgeR_results_QLF_heifer_DESeq_results_Wald_LRT, heifer_DESeq_results_LRT, by.x="Row.names",by.y="row")

heifer_edgeR_results_QLF_heifer_DESeq_results_Wald_LRT<-heifer_edgeR_results_QLF_heifer_DESeq_results_Wald_LRT[with(heifer_edgeR_results_QLF_heifer_DESeq_results_Wald_LRT, order(PValue)),]


heifer_edgeR_results_QLF_heifer_DESeq_results_Wald_LRT[heifer_edgeR_results_QLF_heifer_DESeq_results_Wald_LRT$PValue < 0.002 & heifer_edgeR_results_QLF_heifer_DESeq_results_Wald_LRT$pvalue.x< 0.001 & heifer_edgeR_results_QLF_heifer_DESeq_results_Wald_LRT$pvalue.y < 0.001,  ]
```

```
##               Row.names      logFC   logCPM        F       PValue       FDR
## 1811 ENSBTAG00000004278  0.2149330 5.622078 15.39755 0.0007064172 0.9997511
## 1274 ENSBTAG00000002972 -0.2948188 2.240588 12.99729 0.0015381391 0.9997511
##      baseMean.x log2FoldChange.x    lfcSE.x    stat.x     pvalue.x    padj.x
## 1811  1169.0159        0.2122938 0.05272878  4.026147 5.669837e-05 0.7055545
## 1274   110.8074       -0.2984712 0.08631589 -3.457894 5.444147e-04 0.9998599
##      baseMean.y log2FoldChange.y    lfcSE.y   stat.y     pvalue.y    padj.y
## 1811  1169.0159        0.2122938 0.05272878 16.18059 5.758103e-05 0.7165383
## 1274   110.8074       -0.2984712 0.08631589 11.96050 5.434015e-04 0.9998482
```

```
#heifer_edgeR_results_QLF_heifer_DESeq_results_Wald_LRT_annotated<-merge(heifer_edgeR_results_QLF_heifer_DESeq_results_Wald_LRT, annotation.ensembl.symbol, by.x="Row.names", by.y="ensembl_gene_id", all.x=TRUE, all.y=FALSE)

#write.table(heifer_edgeR_results_QLF_heifer_DESeq_results_Wald_LRT_annotated, "/mnt/storage/lab_folder/shared_R_codes/fernando/angus_holstein_association/results/Additional_file_3.txt",quote = FALSE, sep = "\t",row.names = FALSE, col.names = TRUE)
```

### Calculate q-value - Fertility

```
#Create the permutations matrix
perm_without_replacement <- function(n, r){
 return(factorial(n)/factorial(n - r))
}

permutation_matrix<-matrix(nrow=10000,ncol=22)
#head(permutation_matrix)
dim(permutation_matrix)

for (i in seq(1:10000)){
 sampling <- sample(1:22,22, replace = FALSE)
 if ( ! identical(sampling , c(1:22))){
 permutation_matrix[i,]<-sample(1:22,22, replace = FALSE)
 }}

permutation_matrix<-permutation_matrix[!duplicated(permutation_matrix),]
dim(permutation_matrix)
#head(permutation_matrix)

#permutation_matrix<-permutation_matrix[sample(1:dim(permutation_matrix)[1], 100),]
rand<-dim(permutation_matrix)[1]

sequence.pvalue<-seq(0.0005, 0.002, 0.0001)

#edgeR
results <- filebacked.big.matrix(length(sequence.pvalue),rand, type="double", init=0, separated=FALSE,
 backingfile="incidence_matrix.bin",
 descriptor="incidence_matrix.desc")
mdesc_result<- describe(results)

cl <- makeCluster(30)
registerDoParallel(cl)
results[,]<-foreach(i = sequence.pvalue, .combine='rbind', .inorder=TRUE, .packages=c("edgeR","bigmemory"), .verbose=FALSE) %:%

 foreach(j = 1:rand, .combine='cbind', .inorder=FALSE,.packages=c("edgeR","bigmemory"), .verbose=FALSE ) %dopar%
{

 preg<-factor(heifer_data$preg, levels=c("P", "NP"))
 breed<-factor(heifer_data$breed, levels=c("ANG","HOL"))

 design <- model.matrix(~ breed + preg)
 dds<-DGEList(count=count_data_filtered[,permutation_matrix[j,]], group=heifer_data$preg)
 dds<-estimateDisp(dds, design, robust=TRUE)
 dds <- glmFit(dds, design)
 dds <- glmQLFTest(heifer_QLFit, coef=3)
 length(which(topTags(dds,n=Inf)$table$PValue < i))

 }
stopCluster(cl)
total.rand <- rand * 12065
qvalue<-data.frame(raw.pvalue = sequence.pvalue,
 e.pvalue.edgeR= (rowSums(results[,]+1))/(total.rand+1),
 e.pvalue.edgeR.round= round((rowSums(results[,]+1))/(total.rand+1) ,4))

#qvalue
rm(results)
system("rm incidence_matrix.bin")
system("rm incidence_matrix.desc")

#DESEQ2
results.rand <- filebacked.big.matrix(length(sequence.pvalue),rand, type="double", init=0, separated=FALSE,
 backingfile="incidence_matrix.bin",
 descriptor="incidence_matrix.desc")
mdesc_result<- describe(results.rand)
cl <- makeCluster(30)
registerDoParallel(cl)
results.rand[,]<-foreach(i = sequence.pvalue, .combine='rbind', .inorder=TRUE, .packages=c("DESeq2","bigmemory"), .verbose=FALSE) %:%

 foreach(j = 1:rand, .combine='cbind', .inorder=FALSE,.packages=c("DESeq2","bigmemory"), .verbose=TRUE ) %dopar%
{

heifer_DESeq <-DESeqDataSetFromMatrix(countData=count_data_filtered[permutation_matrix[j,]], colData=heifer_data, design= ~   breed + preg)
heifer_Wald<-DESeq(heifer_DESeq, test="Wald")
heifer_DESeq_results_Wald<-results(heifer_Wald, contrast=c("preg",  "P", "NP"), pAdjustMethod="fdr", tidy=TRUE)

length(which(heifer_DESeq_results_Wald$pvalue < i))

 }
stopCluster(cl)
#results.rand[1:5,1:5]
total.rand <- rand * 12065

qvalue2<-data.frame(raw.pvalue = sequence.pvalue,
 e.pvalue.DESEQ2= (rowSums(results.rand[,]+1))/(total.rand+1),
 e.pvalue.DESEQ2.round= round((rowSums(results.rand[,]+1))/(total.rand+1) ,4))

rm(results.rand)
system("rm incidence_matrix.bin")
system("rm incidence_matrix.desc")


#DESEQ2
results.rand <- filebacked.big.matrix(length(sequence.pvalue),rand, type="double", init=0, separated=FALSE,
 backingfile="incidence_matrix.bin",
 descriptor="incidence_matrix.desc")
mdesc_result<- describe(results.rand)
cl <- makeCluster(30)
registerDoParallel(cl)
results.rand[,]<-foreach(i = sequence.pvalue, .combine='rbind', .inorder=TRUE, .packages=c("DESeq2","bigmemory"), .verbose=FALSE) %:%

 foreach(j = 1:rand, .combine='cbind', .inorder=FALSE,.packages=c("DESeq2","bigmemory"), .verbose=TRUE ) %dopar%
{

heifer_DESeq <-DESeqDataSetFromMatrix(countData=count_data_filtered[permutation_matrix[j,]], colData=heifer_data, design= ~  breed + preg)
heifer_LRT<-DESeq2::DESeq(heifer_DESeq, test="LRT", full= ~ breed + preg, reduced= ~ breed )
heifer_DESeq_results_LRT<-DESeq2::results(heifer_LRT, contrast=c("preg",  "P", "NP"), pAdjustMethod="fdr", tidy=TRUE)

length(which(heifer_DESeq_results_LRT$pvalue < i))

 }
stopCluster(cl)
#results.rand[1:5,1:5]
total.rand <- rand * 12065

qvalue3<-data.frame(raw.pvalue = sequence.pvalue,
 e.pvalue.DESEQ2= (rowSums(results.rand[,]+1))/(total.rand+1),
 e.pvalue.DESEQ2.round= round((rowSums(results.rand[,]+1))/(total.rand+1) ,4))
#qvalue2
qvalue4<-cbind(qvalue,qvalue2,qvalue3)
#qvalue4

rm(results.rand)
system("rm incidence_matrix.bin")
system("rm incidence_matrix.desc")

write.table(qvalue4,file="/mnt/storage/lab_folder/shared_R_codes/fernando/angus_holstein_association/results/2022_10_09_efdr.txt", sep = "\t",append = FALSE, quote = FALSE)
```

## Proteome analysis

### Load and process the data

```
holstein_data<- data.frame(read_excel( "/mnt/storage/lab_folder/shared_R_codes/fernando/angus_holstein_association/resources/220915_Bovine_BS1_01.xlsx"))
angus_data<- data.frame(read_excel( "/mnt/storage/lab_folder/shared_R_codes/fernando/angus_holstein_association/resources/221101_Bovine_Serum.xlsx"))

holstein_data<-holstein_data[holstein_data$Accession %in% angus_data$Accession,]
angus_data<- angus_data[angus_data$Accession %in% holstein_data$Accession,]

proteome_data<-cbind(holstein_data, angus_data)

row.names(proteome_data)<-proteome_data$Accession
#colnames(proteome_data[, c(38:57, 135:158)])
proteome_data<-proteome_data[, c(38:57, 135:158)]
#colnames(proteome_data)

#head(proteome_data)

proteome_data<- proteome_data[rowSums(is.na(proteome_data))<1,]
proteome_data[is.na(proteome_data)]<-0
proteome_data<-log(proteome_data+0.1, base=2)
```

### Analysis with generalized mixed models

```
heifer_data   <- as.data.frame(read_excel("/mnt/storage/lab_folder/shared_R_codes/fernando/angus_holstein_association/resources/sample_info.xlsx"))
preg_factor   <- rep(as.factor(heifer_data$preg), each=2)
breed_factor  <- rep(as.factor(heifer_data$breed), each=2)
subject_factor<-factor(rep(c("A","B","C", "D", "E", "F", "G", "H", "I", "J", "K", "L", "M", "N", "O", "P", "Q", "R", "S", "T", "U", "V"),each=2))
cluster<-rep(as.factor(c("C1","C1","C2","C2","C2","C2","C2","C2","C2","C2","C2","C1","C2","C2","C2","C1","C1","C1","C1","C1","C1","C1")), each=2)
```

```
data_frame_results<-data.frame()

for(i in 1:dim(proteome_data)[1]){
  data_frame_aov<- data.frame(values = t(proteome_data[i,])[,1],groups=preg_factor, breed_factor, subject=subject_factor, cluster= cluster, row.names = NULL)
  model<-lmer(values ~ groups + breed_factor + cluster + (1|subject), data=data_frame_aov)
  anova_results<-Anova(model, type="III", test.statistic="F")
  estimate<-summary(pairs(emmeans(model, "groups", data=data_frame_aov), adjust="none"))
  data_frame_results<-rbind(data_frame_results, data.frame(protein=rownames(proteome_data[i,]),F_stat=anova_results$F[2], p_value=anova_results$`Pr(>F)`[2] ,
                            contrast= estimate$contrast, estimate=estimate$estimate, SE=estimate$SE, p_value_t=estimate$p.value))
}

data_frame_results$fdr<-p.adjust(data_frame_results$p_value, method="fdr")
```

### Analysis with LIMMA

```
design <- model.matrix(~ preg_factor + breed_factor + cluster)
dupcor <- duplicateCorrelation(proteome_data,design,block=subject_factor)

fit <- lmFit(proteome_data, design, block=subject_factor,correlation=dupcor$consensus,method="robust")
fit1 <- eBayes(fit)
results_limma<-topTable(fit1,coef=2,adjust.method="fdr", number=Inf)
```

### Analysis with LIMMA

```
combined_results<-merge(data_frame_results, results_limma, by.x="protein", by.y='row.names')
#combined_results[combined_results$fdr<0.05 & combined_results$adj.P.Val <0.05,]
combined_results<-combined_results[order(combined_results$p_value),]
head(combined_results)
```

```
##     protein    F_stat      p_value contrast   estimate        SE    p_value_t
## 79   A5D798 24.915134 9.456423e-05   NP - P -0.5608937 0.1123696 9.456423e-05
## 159  P19034 18.038222 4.847269e-04   NP - P  1.4345108 0.3377590 4.847269e-04
## 117  F1MYX5 15.618370 9.345866e-04   NP - P  1.1106949 0.2810457 9.345866e-04
## 146  P01035 10.159653 5.101588e-03   NP - P  1.3710125 0.4301322 5.101588e-03
## 120  F1N102  9.784555 5.811597e-03   NP - P  0.7142233 0.2283303 5.811597e-03
## 151  P02768  8.506547 9.209065e-03   NP - P  0.6248395 0.2142357 9.209065e-03
##            fdr      logFC  AveExpr         t      P.Value    adj.P.Val
## 79  0.02014218  0.5874510 32.11571  8.560167 8.677000e-11 1.848201e-08
## 159 0.05162341 -1.3065092 24.79571 -6.039913 3.362501e-07 2.520401e-05
## 117 0.06635565 -1.0872934 25.41215 -5.866786 5.980714e-07 3.184730e-05
## 146 0.24757402 -1.1241175 25.72401 -3.731455 5.605504e-04 7.462327e-03
## 120 0.24757402 -0.7284703 30.61259 -5.713791 9.941039e-07 4.234883e-05
## 151 0.29346188 -0.6415732 31.26988 -4.624993 3.503901e-05 1.203386e-03
##              B
## 79  14.4261528
## 159  6.2480011
## 117  5.6927461
## 146 -0.9985323
## 120  5.1695102
## 151  1.6788828
```

```
#write.table(combined_results, "/mnt/storage/lab_folder/shared_R_codes/fernando/angus_holstein_association/results/Additional_file_5.txt",quote = FALSE, sep = "\t",row.names = FALSE, col.names = TRUE)
```

### Obtain breed specific estimates

#### A5D798 Alpha-ketoglutarate-dependent dioxygenase FTO

```
  data_frame_aov<- data.frame(values = t(proteome_data[rownames(proteome_data)=="A5D798",])[,1],groups=preg_factor, breed_factor, subject=subject_factor, row.names = NULL)
  model<-lmer(values ~ groups*breed_factor + (1|subject), data=data_frame_aov)
  anova_results<-Anova(model, type="III", test.statistic="F")
  model %>% emmeans(pairwise ~ groups | breed_factor)
```

```
## $emmeans
## breed_factor = ANG:
##  groups emmean    SE df lower.CL upper.CL
##  NP       31.2 0.091 18     31.0     31.3
##  P        31.5 0.108 18     31.3     31.8
## 
## breed_factor = HOL:
##  groups emmean    SE df lower.CL upper.CL
##  NP       32.7 0.108 18     32.5     32.9
##  P        33.4 0.108 18     33.2     33.7
## 
## Degrees-of-freedom method: kenward-roger 
## Confidence level used: 0.95 
## 
## $contrasts
## breed_factor = ANG:
##  contrast estimate    SE df t.ratio p.value
##  NP - P     -0.395 0.141 18  -2.798  0.0119
## 
## breed_factor = HOL:
##  contrast estimate    SE df t.ratio p.value
##  NP - P     -0.747 0.152 18  -4.906  0.0001
## 
## Degrees-of-freedom method: kenward-roger
```

#### P19034 Apolipoprotein C-II

```
  data_frame_aov<- data.frame(values = t(proteome_data[rownames(proteome_data)=="P19034",])[,1],groups=preg_factor, breed_factor, subject=subject_factor, row.names = NULL)
  model<-lmer(values ~ groups*breed_factor + (1|subject), data=data_frame_aov)
  anova_results<-Anova(model, type="III", test.statistic="F")
  model %>% emmeans(pairwise ~ groups | breed_factor)
```

```
## $emmeans
## breed_factor = ANG:
##  groups emmean    SE df lower.CL upper.CL
##  NP       24.2 0.250 18     23.7     24.7
##  P        23.7 0.296 18     23.0     24.3
## 
## breed_factor = HOL:
##  groups emmean    SE df lower.CL upper.CL
##  NP       26.9 0.296 18     26.3     27.6
##  P        24.6 0.296 18     24.0     25.2
## 
## Degrees-of-freedom method: kenward-roger 
## Confidence level used: 0.95 
## 
## $contrasts
## breed_factor = ANG:
##  contrast estimate    SE df t.ratio p.value
##  NP - P      0.546 0.388 18   1.410  0.1757
## 
## breed_factor = HOL:
##  contrast estimate    SE df t.ratio p.value
##  NP - P      2.325 0.419 18   5.553  <.0001
## 
## Degrees-of-freedom method: kenward-roger
```

#### F1MYX5 Lymphocyte cytosolic protein 1

```
  data_frame_aov<- data.frame(values = t(proteome_data[rownames(proteome_data)=="F1MYX5",])[,1],groups=preg_factor, breed_factor, subject=subject_factor, row.names = NULL)
  model<-lmer(values ~ groups*breed_factor + (1|subject), data=data_frame_aov)
  anova_results<-Anova(model, type="III", test.statistic="F")
  model %>% emmeans(pairwise ~ groups | breed_factor)
```

```
## $emmeans
## breed_factor = ANG:
##  groups emmean    SE df lower.CL upper.CL
##  NP       27.2 0.218 18     26.7     27.6
##  P        26.7 0.258 18     26.2     27.3
## 
## breed_factor = HOL:
##  groups emmean    SE df lower.CL upper.CL
##  NP       24.5 0.258 18     23.9     25.0
##  P        22.6 0.258 18     22.1     23.1
## 
## Degrees-of-freedom method: kenward-roger 
## Confidence level used: 0.95 
## 
## $contrasts
## breed_factor = ANG:
##  contrast estimate    SE df t.ratio p.value
##  NP - P      0.429 0.337 18   1.270  0.2202
## 
## breed_factor = HOL:
##  contrast estimate    SE df t.ratio p.value
##  NP - P      1.855 0.365 18   5.089  0.0001
## 
## Degrees-of-freedom method: kenward-roger
```

## Multi-omics analysis

### Preparation of the data for analysis

```
vst_DESeq <- DESeq2::vst( DESeq2::DESeqDataSetFromMatrix(countData=count_data_filtered, colData=sample_info, design= ~ preg + breed), blind = TRUE, nsub = 5000)
vst_DESeq_data<-assay(vst_DESeq)
colnames(vst_DESeq_data)<-heifer_data$SNP_array_ID


vst_DESeq_data<-vst_DESeq_data[rownames(vst_DESeq_data) %in%
heifer_edgeR_results_QLF_heifer_DESeq_results_Wald_LRT[heifer_edgeR_results_QLF_heifer_DESeq_results_Wald_LRT$PValue < 0.01 & heifer_edgeR_results_QLF_heifer_DESeq_results_Wald_LRT$pvalue.x< 0.01 & heifer_edgeR_results_QLF_heifer_DESeq_results_Wald_LRT$pvalue.y < 0.01,  1] ,]

colnames(proteome_data)<-rep(heifer_data$SNP_array_ID, each=2)
proteome_data_mofa<-proteome_data
proteome_data_mofa$proteins<-rownames(proteome_data_mofa)
proteome_data_mofa<-reshape2::melt(proteome_data_mofa)
proteome_data_mofa<-data.frame(proteome_data_mofa %>% group_by(proteins, variable) %>% dplyr::summarize(Mean = mean(value, na.rm=TRUE)), stringsAsFactors = FALSE)
proteome_data_mofa$variable<-as.character(proteome_data_mofa$variable)
proteome_data_mofa<-proteome_data_mofa[!duplicated(proteome_data_mofa[,2:3]),]
proteome_data_mofa<-reshape2::dcast(proteome_data_mofa, proteins ~ variable, value.var ="Mean")
rownames(proteome_data_mofa)<-proteome_data_mofa$proteins
proteome_data_mofa<-proteome_data_mofa[,heifer_data$SNP_array_ID]

proteome_data_mofa<-proteome_data_mofa[rownames(proteome_data_mofa) %in% combined_results[combined_results$p_value<0.05 & combined_results$P.Value<0.05,1],]

holstein_data<- data.frame(read_excel( "/mnt/storage/lab_folder/shared_R_codes/fernando/angus_holstein_association/resources/220915_Bovine_BS1_01.xlsx"))
angus_data<- data.frame(read_excel( "/mnt/storage/lab_folder/shared_R_codes/fernando/angus_holstein_association/resources/221101_Bovine_Serum.xlsx"))
holstein_data<-holstein_data[holstein_data$Accession %in% angus_data$Accession,]
angus_data<- angus_data[angus_data$Accession %in% holstein_data$Accession,]
proteome_data_annotation<-cbind(holstein_data, angus_data)
proteome_data_annotation<-proteome_data_annotation[, c("Accession", "Gene.Symbol")]

proteome_data_mofa<-merge(proteome_data_mofa,proteome_data_annotation, by.x="row.names", by.y="Accession")


ah_genotypes<-data.table::fread("/mnt/storage/lab_folder/heifer_infertility/AI_angus_holstein/assoc_analysis/Virginia_Tech_Univ_Biase_BOV770V01_20220825_FinalReport.txt", header = TRUE, sep = "\t", skip = 9, check.names=TRUE)

ah_genotypes<-ah_genotypes[ah_genotypes$SNP.Name %in% angus_holstein_genotypes_qc_data.fisher[angus_holstein_genotypes_qc_data.fisher$P<0.001,2],]

angus_holstein_genotypes<- ah_genotypes[,c(1,2,7,8 )]
angus_holstein_genotypes$genotype <- paste(angus_holstein_genotypes$Allele1...AB, angus_holstein_genotypes$Allele2...AB, sep="")

angus_holstein_genotypes<- angus_holstein_genotypes[,-c(3,4)]

#head(angus_holstein_genotypes)
angus_holstein_genotypes<-angus_holstein_genotypes[!(angus_holstein_genotypes$Sample.ID %in% c("Sample 23", "Sample 24"))]

angus_holstein_genotypes_a<- as.data.frame(tidyr::spread(angus_holstein_genotypes, Sample.ID, genotype))

rownames(angus_holstein_genotypes_a)<- angus_holstein_genotypes_a$SNP.Name
angus_holstein_genotypes_a<- angus_holstein_genotypes_a[,-1]

colnames(angus_holstein_genotypes_a)<-make.names(colnames(angus_holstein_genotypes_a))

angus_holstein_genotypes_a[angus_holstein_genotypes_a=="AA"]<-as.numeric(0)
angus_holstein_genotypes_a[angus_holstein_genotypes_a=="AB"]<-as.numeric(1)
angus_holstein_genotypes_a[angus_holstein_genotypes_a=="BB"]<-as.numeric(1)

angus_holstein_genotypes_b<-angus_holstein_genotypes_a[rowSums(angus_holstein_genotypes_a=='1',na.rm=TRUE)>=4,]
angus_holstein_genotypes_b<-angus_holstein_genotypes_a[rowSums(angus_holstein_genotypes_a=='2',na.rm=TRUE)>=4,]
angus_holstein_genotypes_b<-angus_holstein_genotypes_a[rowSums(angus_holstein_genotypes_a=='0',na.rm=TRUE)>=4,]

angus_holstein_genotypes_b1<-data.frame(lapply(angus_holstein_genotypes_b,as.numeric))
rownames(angus_holstein_genotypes_b1)<- rownames(angus_holstein_genotypes_b)

angus_holstein_genotypes_b1<-as.matrix(angus_holstein_genotypes_b1,rownames = TRUE)

colnames(angus_holstein_genotypes_b1)<-tolower(gsub(".", "_", colnames(angus_holstein_genotypes_b1),fixed = TRUE))

genotypes_b <- angus_holstein_genotypes_b1[,heifer_data$SNP_array_ID]

snp_map_genotypes <- read.table("/mnt/storage/lab_folder/heifer_infertility/AI_angus_holstein/assoc_analysis/SNP_Map.txt", header = TRUE, sep = "\t")
snp_map_genotypes <- snp_map_genotypes[,c(3,2,4)]

snp_map_genotypes<- snp_map_genotypes[!snp_map_genotypes$Chromosome=="0" & !snp_map_genotypes$Chromosome=="X" & !snp_map_genotypes$Chromosome=="Y",]

genotypes_b<-genotypes_b[rownames(genotypes_b) %in% snp_map_genotypes$Name,]

mofa_list_matrix<-list(mRNA=as.matrix(vst_DESeq_data), protein=as.matrix(proteome_data_mofa), genotypes=as.matrix(genotypes_b))

covariates_mofa<-data.frame(row.names=heifer_data$SNP_array_ID, breed=heifer_data$breed, pregnancy=heifer_data$preg)
covariates_mofa$pregnancy<-ifelse(covariates_mofa$pregnancy == "P", "Fertile", "Sub-fertile")
covariates_mofa$breed<-ifelse(covariates_mofa$breed == "HOL", "Holstein", "Angus")
```

### Organize objects and run analysis

```
multiomics_mofa <- MultiAssayExperiment(
  experiments = mofa_list_matrix, 
  colData = covariates_mofa
)

MOFAobject <- create_mofa_from_MultiAssayExperiment(multiomics_mofa,extract_metadata = TRUE, groups = "breed")

data_opts <- get_default_data_options(MOFAobject)
head(data_opts)
model_opts <- get_default_model_options(MOFAobject)
head(model_opts)
model_opts$likelihoods<-c("gaussian" ,"gaussian", "bernoulli" )
head(model_opts)
train_opts <- get_default_training_options(MOFAobject)
head(train_opts)
train_opts$convergence_mode<-"slow"

set.seed(12345)

MOFAobject <- prepare_mofa(
  object = MOFAobject,
  data_options = data_opts,
  model_options = model_opts,
  training_options = train_opts
)

MOFAobject.trained.group <- run_mofa(MOFAobject, outfile = "/mnt/storage/lab_folder/shared_R_codes/fernando/angus_holstein_association/results/MOFAobject.trained_2022_11_28_group.hdf5", save_data = TRUE, use_basilisk = TRUE)
```

### Load the model

```
MOFAobject.trained.group<-load_model("/mnt/storage/lab_folder/shared_R_codes/fernando/angus_holstein_association/results/MOFAobject.trained_2022_11_28_group.hdf5", remove_inactive_factors = TRUE)

head(MOFAobject.trained.group@cache$variance_explained$r2_total[[1]])
```

```
##      mRNA   protein genotypes 
##  44.12020  16.55246  70.13337
```

```
head(MOFAobject.trained.group@cache$variance_explained$r2_per_factor[[1]], n=10)
```

```
##                 mRNA     protein    genotypes
## Factor1  0.005228172  0.02095313 64.481634390
## Factor2 44.102950596  3.60692713  0.008542051
## Factor3  0.006075510 12.91058002  0.001576960
## Factor4  0.005947563  0.01400409  5.641619256
```

## Figures

### Figure 1

```
evec <- data.table::fread("/mnt/storage/lab_folder/heifer_infertility/AI_angus_holstein/assoc_analysis/angus_holstein_genotypes_qc_data.eigenvec", data.table = FALSE)
eval <- data.table::fread("/mnt/storage/lab_folder/heifer_infertility/AI_angus_holstein/assoc_analysis/angus_holstein_genotypes_qc_data.eigenval", data.table = FALSE)

percentage_PCA1<-round((eval$V1[1] / sum(eval$V1) )*100 ,2)
percentage_PCA2<-round((eval$V1[2] / sum(eval$V1) )*100 ,2)

plot_1<-ggplot(evec) + 
  geom_point(aes(V3, V4, shape=factor(rep(c("Holstein","Angus"),c(10,12))), color=factor(c("NP","NP","NP","NP","NP","P","P","P","P","P","NP","NP","NP","NP","NP","NP","NP","P","P","P","P","P"))), size=3)+
  scale_shape_manual(values=c("Holstein"= 0,"Angus"=2))+
  scale_color_manual(values=c("P"="#0066b9", "NP"= "#ff7176"))+
  labs(x = paste("PC1: ",percentage_PCA1,"% variance", sep=""), y = paste("PC2: ",percentage_PCA2,"% variance", sep=""))+
  ggtitle("PCA SNPs")+
  theme_minimal(base_size = 12)+
 theme(
    axis.text = element_blank(),
    plot.margin=grid::unit(c(0,0,0,0), "mm"),
    legend.position = "bottom",
    legend.title = element_text(size=10),
    legend.text  = element_text(size=10),
    plot.title = element_text(hjust = 0.5, size=12)
  )
```

```
vst_DESeq <- DESeq2::vst( DESeq2::DESeqDataSetFromMatrix(countData=count_data_filtered, colData=sample_info, design= ~ preg + breed), blind = TRUE, nsub = 5000)

pcaData<-plotPCA(vst_DESeq, intgroup=c("preg", "breed"), returnData=TRUE)
percentVar <- round(100 * attr(pcaData, "percentVar"))

plot_2<-ggplot(pcaData, aes(PC1, PC2, color=preg, shape= breed)) +
  scale_shape_manual(name=NULL, values=c("HOL"= 2,"ANG"=0), labels=c("Holstein", "Angus"))+
  scale_color_manual(name=NULL, values=c("P" = "#0066b9","NP"= "#ff7176"), labels=c("Fertile", "Sub-fertile"))+
  geom_point(size=3) +
  xlab(paste0("PC1: ",percentVar[1],"% variance")) +
  ylab(paste0("PC2: ",percentVar[2],"% variance")) + 
  ggtitle("PCA transcriptome data")+
  theme_minimal(base_size = 12)+
 theme(
    axis.text = element_blank(),
    plot.margin=grid::unit(c(0,0,0,0), "mm"),
    legend.position = "bottom",
    legend.title = element_text(size=10),
    legend.text  = element_text(size=10),
    plot.title = element_text(hjust = 0.5, size=12)
  )
legend<-get_legend( plot_2 )
```

```
colnames(proteome_data)<-rep(heifer_data$SNP_array_ID, each=2)
proteome_data_pca<-proteome_data
proteome_data_pca$proteins<-rownames(proteome_data_pca)
proteome_data_pca<-reshape2::melt(proteome_data_pca)
proteome_data_pca<-data.frame(proteome_data_pca %>% group_by(proteins, variable) %>% dplyr::summarize(Mean = mean(value, na.rm=TRUE)), stringsAsFactors = FALSE)
proteome_data_pca$variable<-as.character(proteome_data_pca$variable)
proteome_data_pca<-proteome_data_pca[!duplicated(proteome_data_pca[,2:3]),]
proteome_data_pca<-reshape2::dcast(proteome_data_pca, proteins ~ variable)

pca_proteome<- prcomp(na.omit(proteome_data_pca[,c(2:23)]))

summary_PCA<-summary(pca_proteome)
PCA1<-round(summary_PCA$importance[2,1]*100,1)
PCA2<-round(summary_PCA$importance[2,2]*100,1)

pca_proteome<-cbind(pca_proteome$rotation,heifer_data)

plot_3<-ggplot(pca_proteome, aes(x=PC1, y=PC2, color=preg, shape= breed)) +
  scale_shape_manual(name=NULL, values=c("HOL"= 2,"ANG"=0), labels=c("Holstein", "Angus"))+
  scale_color_manual(name=NULL, values=c("P" = "#0066b9","NP"= "#ff7176"), labels=c("Fertile", "Sub-fertile"))+
  geom_point(size=3) +
  xlab(paste0("PC1: ",PCA1,"% variance")) +
  ylab(paste0("PC2: ",PCA2,"% variance")) + 
  ggtitle("PCA proteome data")+
  theme_minimal(base_size = 12)+
 theme(
    axis.text = element_blank(),
    plot.margin=grid::unit(c(0,0,0,0), "mm"),
    legend.position = "bottom",
    legend.title = element_text(size=10),
    legend.text  = element_text(size=10),
    plot.title = element_text(hjust = 0.5, size=12))
  # + geom_text_repel(aes(label = pca_proteome$sample))
```

```
fig <- ggdraw() + draw_image(magick::image_read_pdf("/mnt/storage/lab_folder/shared_R_codes/fernando/angus_holstein_association/Fig1_A_B.pdf", density = 600))
cowplot::plot_grid(
  cowplot::plot_grid(fig),
  NULL,
 cowplot::plot_grid(cowplot::plot_grid(plot_1+ theme(legend.position="none"), NULL, plot_2+ theme(legend.position="none"), NULL, plot_3+ theme(legend.position="none") , rel_widths = c(1,0.1,1,0.1,1), nrow=1, labels=c("C", "", "D","","E"),label_fontface = "plain", label_size=12),
                   legend, nrow=2, rel_heights = c(1,0.1)),
                   rel_heights = c(0.8,0.05,0.8), nrow=3)
```

```
pdf(file="/mnt/storage/lab_folder/shared_R_codes/fernando/angus_holstein_association/results/figure_1.pdf", width=6.69, height=4.7)

fig <- ggdraw() + draw_image(magick::image_read_pdf("/mnt/storage/lab_folder/shared_R_codes/fernando/angus_holstein_association/Fig1_A_B.pdf", density = 600))
cowplot::plot_grid(
  cowplot::plot_grid(fig),
  NULL,
 cowplot::plot_grid(cowplot::plot_grid(plot_1+ theme(legend.position="none"), NULL, plot_2+ theme(legend.position="none"), NULL, plot_3+ theme(legend.position="none") , rel_widths = c(1,0.1,1,0.1,1), nrow=1, labels=c("C", "", "D","","E"),label_fontface = "plain", label_size=12),
                   legend, nrow=2, rel_heights = c(1,0.1)),
                   rel_heights = c(0.8,0.05,0.8), nrow=3)
                   
dev.off()
```

### Figure 2

```
angus_holstein_genotypes_qc_data.fisher$CHR<-paste("chr",angus_holstein_genotypes_qc_data.fisher$CHR, sep="")
angus_holstein_genotypes_qc_data.fisher.GRanges<-GenomicRanges::makeGRangesFromDataFrame(angus_holstein_genotypes_qc_data.fisher, ignore.strand=TRUE,seqinfo=NULL,seqnames.field=c("CHR"),  start.field="BP", end.field=c("BP"),keep.extra.columns=TRUE)
ch<-rtracklayer::import.chain("/home/fbiase/genome/lifover_chains/bosTau8ToBosTau9.over.chain")
seqlevelsStyle(angus_holstein_genotypes_qc_data.fisher.GRanges) <-"UCSC"
angus_holstein_genotypes_qc_data.fisher.GRanges_btau9 <- rtracklayer::liftOver(angus_holstein_genotypes_qc_data.fisher.GRanges, ch)
#angus_holstein_genotypes_qc_data.fisher.GRanges_btau9
angus_holstein_genotypes_qc_data.fisher.GRanges_btau9 <- unlist(angus_holstein_genotypes_qc_data.fisher.GRanges_btau9)
genome(angus_holstein_genotypes_qc_data.fisher.GRanges_btau9) <- "btau9"
#angus_holstein_genotypes_qc_data.fisher.GRanges_btau9
angus_holstein_genotypes_qc_data.fisher.GRanges_btau9_df<- data.frame(iranges = angus_holstein_genotypes_qc_data.fisher.GRanges_btau9) 
#write.table(angus_holstein_genotypes_qc_data.fisher.GRanges_btau9_df, "/mnt/storage/lab_folder/shared_R_codes/fernando/angus_holstein_association/results/Additional_file_3.txt",quote = FALSE, sep = "\t",row.names = FALSE, col.names = TRUE)
angus_holstein_genotypes_qc_data.fisher.GRanges_btau9_df$label<-""
angus_holstein_genotypes_qc_data.fisher.GRanges_btau9_df$label<-ifelse(angus_holstein_genotypes_qc_data.fisher.GRanges_btau9_df$iranges.P <= 2.658e-05, paste(angus_holstein_genotypes_qc_data.fisher.GRanges_btau9_df$iranges.seqnames,":", angus_holstein_genotypes_qc_data.fisher.GRanges_btau9_df$iranges.start, sep="") ,"")
angus_holstein_genotypes_qc_data.fisher.GRanges_btau9_df$iranges.seqnames<-str_replace(angus_holstein_genotypes_qc_data.fisher.GRanges_btau9_df$iranges.seqnames,"chr","")
  
plot_3<-ggmanh::manhattan_plot(x = angus_holstein_genotypes_qc_data.fisher.GRanges_btau9_df, pval.colname = "iranges.P", chr.colname = "iranges.seqnames", pos.colname = "iranges.start",  rescale = FALSE, signif = c( 1e-05), label.font.size = 6 , force = 20, chr.order =c("1", "2", "3","4","5" , "6" , "7",  "8" , "9", "10", "11", "12", "13", "14", "15", "16", "17", "18", "19", "20", "21", "22", "23" ,"24", "25" ,"26" ,"27", "28", "29" ),label.colname ="label")
```

```
ah_genotypes<- data.table::fread("/mnt/storage/lab_folder/heifer_infertility/AI_angus_holstein/assoc_analysis/Virginia_Tech_Univ_Biase_BOV770V01_20220825_FinalReport.txt", header = TRUE, sep = "\t", skip = 9)
ah_genotypes<-ah_genotypes[,c(1,2,3,4)]
colnames(ah_genotypes)<-c("SNP_Name", "Sample_ID","Allele1", "Allele2")
ah_genotypes<-ah_genotypes[ah_genotypes$SNP_Name %in% c("BovineHD1200026258","Hapmap31720-BTA-126418"),]
ah_genotypes$Sample_ID <- gsub(" ","_",ah_genotypes$Sample_ID)
ah_genotypes<- ah_genotypes[!ah_genotypes$Sample_ID=="Sample_23" & !ah_genotypes$Sample_ID=="Sample_24",]
ah_genotypes$genotype<-paste(ah_genotypes$Allele1,ah_genotypes$Allele2, sep="")
#ah_genotypes %>% group_by(SNP_Name, genotype) %>% tally()
sample_id <- c("Sample_1","Sample_2","Sample_3","Sample_4","Sample_5","Sample_6","Sample_7","Sample_8","Sample_9","Sample_10","Sample_11","Sample_12","Sample_13","Sample_14","Sample_15","Sample_16","Sample_17","Sample_18","Sample_19","Sample_20","Sample_21","Sample_22") 
breed<-c("hosltein","hosltein","hosltein","hosltein","hosltein","hosltein","hosltein","hosltein","hosltein","hosltein","angus","angus","angus","angus","angus","angus","angus","angus","angus","angus","angus","angus")
phenotype_angus_holstein <- c("2","2","2","2","2","1","1","1","1","1","2","2","2","2","2", "2","2","1","1","1","1","1")
ah_genotypes<-merge(ah_genotypes,data.frame(sample_id,phenotype_angus_holstein, breed), by.x="Sample_ID", by.y="sample_id")
ah_genotypes<-ah_genotypes[!(ah_genotypes$genotype == "--"),]
ah_genotypes_count<-ah_genotypes %>% group_by(SNP_Name, genotype,phenotype_angus_holstein) %>% tally() %>% arrange(phenotype_angus_holstein, .by_group = TRUE )

supp.labs <- c("rs110918927", "rs109366560")
names(supp.labs) <- c("BovineHD1200026258", "Hapmap31720-BTA-126418")

plot_4<-ggplot(ah_genotypes_count, aes(fill= genotype , y=n, x=phenotype_angus_holstein))+
geom_bar(position="fill", stat="identity")+
scale_fill_manual(values=c("AA" = "#cb6054","AG"= "#87a14d", "GG"="#9c72be"))+
scale_y_continuous(name="Frequency")+
scale_x_discrete(name=NULL, labels=c("Fertile", "Sub-fertile"))+
facet_grid(~SNP_Name, labeller = labeller(SNP_Name = supp.labs))+
    theme_classic(base_size=12)+
    theme(
    axis.text.x=element_text(color="black", angle=45, hjust=1),
    axis.text.y=element_text(color="black"),
    strip.background = element_blank(),
    strip.text =  element_text(color="black",  size=8),
    legend.title=element_blank(),
    legend.position="bottom",
    legend.key.size = unit(3, 'mm')
)
```

```
plot_grid(plot_3,plot_4, nrow=1, rel_widths=c(0.6,0.3))
```

```
pdf(file="/mnt/storage/lab_folder/shared_R_codes/fernando/angus_holstein_association/results/figure_2.pdf", width=6.69, height=3)
plot_grid(plot_3,plot_4, nrow=1, rel_widths=c(0.6,0.3))
dev.off()
```

### Figure 3

```
plotdata <- data_cpm_filtered
plotdata <- plotdata[rownames(plotdata)=="ENSBTAG00000004278",]
plotdata <- as.data.frame(plotdata)
plotdata <- cbind(heifer_data, plotdata)
plotdata$shape <- c(0:9, 0:11)

plotdata2 <- data_cpm_filtered
plotdata2 <- plotdata2[rownames(plotdata2)=="ENSBTAG00000002972",]
plotdata2 <- as.data.frame(plotdata2)
plotdata2 <- cbind(heifer_data, plotdata2)
plotdata2$shape <- c(0:9, 0:11)


plotdata %>% group_by(breed, preg) %>% dplyr::summarize(Mean = mean(plotdata, na.rm=TRUE))
```

```
## `summarise()` has grouped output by 'breed'. You can override using the
## `.groups` argument.
```

```
## # A tibble: 4 × 3
## # Groups:   breed [2]
##   breed preg   Mean
##   <chr> <chr> <dbl>
## 1 ANG   NP     49.9
## 2 ANG   P      55.1
## 3 HOL   NP     40.7
## 4 HOL   P      50.1
```

```
plotdata2 %>% group_by(breed, preg) %>% dplyr::summarize(Mean = mean(plotdata2, na.rm=TRUE))
```

```
## `summarise()` has grouped output by 'breed'. You can override using the
## `.groups` argument.
```

```
## # A tibble: 4 × 3
## # Groups:   breed [2]
##   breed preg   Mean
##   <chr> <chr> <dbl>
## 1 ANG   NP     4.44
## 2 ANG   P      3.64
## 3 HOL   NP     5.85
## 4 HOL   P      4.73
```

```
font_size=14
plot_5 <- ggplot(data=plotdata, aes(y=plotdata, x=breed, color=preg))+
geom_point(position=position_jitterdodge(jitter.width = 0.5,jitter.height = 0,dodge.width = 1),size=2, shape=plotdata$shape)+
  scale_color_manual(labels=c("Sub-fertile", "Fertile"), values=c("#ff7176", "#0066b9"))+
  scale_x_discrete(name =NULL, labels=c("Angus", "Holstein"))+
  ylab("CPM")+
  labs(title = expression(""*italic("APMAP")),subtitle = expression("adipocyte plasma membrane associated protein"))+
  theme_classic2()+
  theme(plot.title = element_text(size=14, face = "italic"),
        plot.subtitle = element_text(size=10, face = "italic"),
        legend.key = element_rect(),
        legend.position = "none",
        axis.text.x = element_text(size=font_size, color="black"),
        axis.text.y = element_text(size=font_size, color="black"),
        axis.title.y = element_text(size=font_size, color="black"),
        axis.title.x = element_text(size=font_size, color="black"))

plot_6 <- ggplot(data=plotdata2, aes(y=plotdata2, x=breed, color=preg))+
geom_point(position=position_jitterdodge(jitter.width = 0.5,jitter.height = 0,dodge.width = 1),size=2, shape=plotdata$shape)+
  scale_color_manual(labels=c("Sub-fertile", "Fertile"), values=c("#ff7176", "#0066b9"))+
  scale_x_discrete(name =NULL, labels=c("Angus", "Holstein"))+
  ylab("")+
  labs(title = expression(""*italic("DNAI7")),subtitle = expression("dynein axonemal intermediate chain 7"))+
  theme_classic2()+
  theme(plot.title = element_text(size=14, face = "italic"),
        plot.subtitle = element_text(size=10, face = "italic"),
        legend.key = element_rect(),
        legend.position = "bottom",
        legend.title = element_blank(),
        legend.text = element_text(size=font_size, color="black"),
        axis.text.x = element_text(size=font_size, color="black"),
        axis.text.y = element_text(size=font_size, color="black"),
        axis.title.y = element_text(size=font_size, color="black"),
        axis.title.x = element_text(size=font_size, color="black"))
legend <- get_legend(plot_6)


#plots <- plot_grid(plot_5, plot_6+theme(legend.position="none"), nrow=1)
#plot_grid(plots, legend, nrow=2, ncol=1, rel_heights = c(1, .1))

colnames(proteome_data)<-c("07","07","08","08","09","09","10","10","11","11","12","12","13","13","14","14","15","15","16","16","17","17","18","18","19","19","20","20","21","21","22","22","23","23","24","24","25","25","26","26","27","27","28","28")
heifer_data <- sample_info

plotdata <- proteome_data
plotdata <- plotdata[rownames(plotdata)=="A5D798",]
plotdata <- reshape2::melt(plotdata)
```

```
## No id variables; using all as measure variables
```

```
plotdata <- merge(heifer_data, plotdata, by.x="sample", by.y="variable")

plotdata<- plotdata %>% group_by(breed, preg,Animal_ID) %>% dplyr::summarize(Mean = mean(value, na.rm=TRUE))
```

```
## `summarise()` has grouped output by 'breed', 'preg'. You can override using the
## `.groups` argument.
```

```
plotdata$shape <- c(c(0,1,2,3,4,5,6,7,8,9),
                    c(0,1,2,3,4,5,6,7,8,9,10,11))

font_size=10

 plot_7<-ggplot(data=plotdata, aes(y=Mean, x=breed, color=preg))+
  geom_point(position=position_jitterdodge(jitter.width = 0.5,jitter.height = 0,dodge.width = 1),size=2, shape=plotdata$shape)+
  scale_color_manual(labels=c("Sub-fertile", "Fertile"), values=c("#ff7176", "#0066b9"))+
  scale_x_discrete(name =NULL, labels=c("Angus", "Holstein"))+
  ylab("Log(Protein abundance)")+
  labs(title = expression("FTO"),subtitle = expression("Alpha-ketoglutarate-dependent dioxygenase FTO"))+
  theme_classic2()+
  annotate("text", x = c(0.9,2), y = c(32.5,32), label = c("estimate = -0.395\nP = 0.0119","estimate = -0.747\nP = 0.0001"),size=3)+
  theme(legend.key = element_rect(),
        legend.position = "bottom", 
        legend.title = element_blank(),
        plot.title = element_text(size=14, face = "italic"),
        plot.subtitle = element_text(size=10, face = "italic"),
        axis.text.x = element_text(size=font_size, color="black"),
        axis.text.y = element_text(size=font_size, color="black"),
        axis.title.y = element_text(size=font_size, color="black"),
        axis.title.x = element_text(size=font_size, color="black"))
```

```
plot_grid(
plot_grid( plot_grid(plot_5, plot_6+theme(legend.position="none") , nrow=1), plot_grid(plot_7+theme(legend.position="none"), nrow=1),labels=c("A","B"),label_fontface = "plain",rel_widths = c(1,0.6)),legend, nrow=2, ncol=1, rel_heights = c(1, .1)
)
```

```
plot_grid(
plot_grid( plot_grid(plot_5, plot_6+theme(legend.position="none") , nrow=1),labels=c("A","B"),label_fontface = "plain",rel_widths = c(1,0.6)),legend, nrow=2, ncol=1, rel_heights = c(1, .1)
)
```

pdf(file=“/mnt/storage/lab\_folder/shared\_R\_codes/fernando/angus\_holstein\_association/results/figure\_3.pdf”,
width=6.69, height=3) plot\_grid( plot\_grid( plot\_grid(plot\_5,
plot\_6+theme(legend.position=“none”) , nrow=1),
plot\_grid(plot\_7+theme(legend.position=“none”),
nrow=1),labels=c(“A”,“B”),label\_fontface = “plain”,rel\_widths =
c(1,0.6)),legend, nrow=2, ncol=1, rel\_heights = c(1, .1) ) dev.off()

### Figure 4

```
plot_10<-plot_variance_explained(MOFAobject.trained.group, x="view", y="factor")+
theme(axis.text.x=element_text(size=7,angle=60,vjust=1,hjust=1),
      axis.text.y=element_text(size=7),
      plot.title=element_text(size=7),
      strip.text = element_text(size = 7),
      legend.position="top",
      legend.text = element_text(size=7),
      legend.title = element_text(size=7),
      legend.key.height = unit(4,units="mm"),
      plot.margin=unit(x=c(0,3,0,0),units="mm"),
      legend.margin=margin(c(0,10,0,0), unit='mm'))


plot_11<-plot_factor(MOFAobject.trained.group, 
  factor = 1:3,
  color_by = "pregnancy",
  shape_by = "breed"
) +
scale_fill_manual(name=NULL, values=c("Fertile" = "#0066b9","Sub-fertile"= "#ff7176"), labels=c("Fertile", "Sub-fertile"))+
scale_shape_manual(name=NULL, values=c(21,23))+
theme(axis.text.y=element_text(size=7),
      axis.text.x=element_text(size=7,angle=60,vjust=1,hjust=1),
      strip.text = element_text(size = 7),
      legend.position="bottom",
      legend.spacing.x = unit(0, 'cm'),
      legend.text=element_text(size=6),
      legend.key.size = unit(2, 'mm'))
```

```
## Scale for shape is already present.
## Adding another scale for shape, which will replace the existing scale.
```

```
body(plot_top_weights)[[22]][[3]][[2]][[3]][[3]][[4]]<-1

plot_12<-plot_top_weights(MOFAobject.trained.group,
  view = "genotypes",
  factor = c(1:3),
  nfeatures = 10)+
  theme(
axis.title.x=element_blank(),
axis.text.y = element_text(size = 6),
axis.text.x = element_text(size = 6),
strip.text = element_text(size = 6),
plot.margin=unit(x=c(t = 0, r = 0, b = 0, l = 0),units="mm")
)

plot_13<-plot_top_weights(MOFAobject.trained.group,
  view = "mRNA",
  factor = c(1:3),
  nfeatures = 10)+
  theme(
axis.title.x=element_blank(),
axis.text.y = element_text(size = 6),
axis.text.x = element_text(size = 6),
strip.text = element_text(size = 6),
plot.margin=unit(x=c(t = 0, r = 0, b = 0, l = 0),units="mm")
)

plot_14<-plot_top_weights(MOFAobject.trained.group,
  view = "protein",
  factor = c(1:3),
  nfeatures = 10)+
  theme(
axis.title.x=element_blank(),
axis.text.y = element_text(size = 6),
axis.text.x = element_text(size = 6),
strip.text = element_text(size = 6),
plot.margin=unit(x=c(t = 0, r = 0, b = 0, l = 14),units="mm"),
panel.spacing = unit(c(4,3), "lines")
)


MOFAobject.trained.group <- run_tsne(MOFAobject.trained.group,perplexity = 5)

plot_15<-plot_dimred(MOFAobject.trained.group,
  method = "TSNE",  # method can be either "TSNE" or "UMAP"
  dot_size=3,
  color_by = "pregnancy",
  shape_by = "breed"
)+
scale_fill_manual(values=c("Fertile" = "#0066b9","Sub-fertile"= "#ff7176"), labels=c("Fertile", "Sub-fertile"))+
ggtitle("t-Distributed Stochastic \n Neighbor Embedding")+
theme(legend.position="none",
plot.title = element_text(size=10, hjust=0.5))
```

```
## Warning: The `<scale>` argument of `guides()` cannot be `FALSE`. Use "none" instead as
## of ggplot2 3.3.4.
## ℹ The deprecated feature was likely used in the MOFA2 package.
##   Please report the issue at <https://github.com/bioFAM/MOFA2>.
## This warning is displayed once every 8 hours.
## Call `lifecycle::last_lifecycle_warnings()` to see where this warning was
## generated.
```

```
cowplot::plot_grid(
cowplot::plot_grid(plot_10,plot_11, plot_15,nrow=3, labels = c("A","B","D"),label_size = 13, label_fontface = "plain"),
cowplot::plot_grid(cowplot::plot_grid(plot_12,plot_13, plot_14, nrow=3, rel_heights=c(0.9,0.9,1)),labels = c("C"),label_size = 13, label_fontface = "plain"), 
ncol=2, rel_widths=c(0.4,1))
```

pdf(file=“/mnt/storage/lab\_folder/shared\_R\_codes/fernando/angus\_holstein\_association/results/figure\_4.pdf”,
width=6.69, height=6.5) cowplot::plot\_grid(
cowplot::plot\_grid(plot\_10,plot\_11, plot\_15,nrow=3, labels =
c(“A”,“B”,“D”),label\_size = 13, label\_fontface = “plain”),
cowplot::plot\_grid(cowplot::plot\_grid(plot\_12,plot\_13, plot\_14, nrow=3,
rel\_heights=c(0.9,0.9,1)),labels = c(“C”),label\_size = 13,
label\_fontface = “plain”), ncol=2, rel\_widths=c(0.4,1)) dev.off()

### plot eFDR

```
efdr<-read.delim("/mnt/storage/lab_folder/shared_R_codes/fernando/angus_holstein_association/results/2022_10_09_efdr.txt")
font_size<-9

plot1<-ggplot()+
geom_point(data=efdr, aes(x=e.pvalue.edgeR , y=raw.pvalue),color="black", size=2,shape=16)+
geom_line(data=efdr, aes(x=e.pvalue.edgeR , y=raw.pvalue),color="black", size=0.1,linetype=3)+
scale_x_continuous(name="empirical FDR", limits = c(0, 0.0005), breaks=seq(0, 0.0005, 0.0001))+
scale_y_continuous(name="nominal P value", limits = c(0, 0.002), breaks=seq(0, 0.002, 0.0005))+
ggtitle("Differential gene expression eFDR \n edgeR")+
theme_bw()+
theme(panel.grid= element_blank(),
panel.background = element_blank(),
panel.grid.minor = element_blank(),
panel.grid.major = element_line(color="lightgray"),
plot.background = element_blank(),
axis.text.y=element_text(color="black", size=font_size),
axis.text.x=element_text(color="black", size=font_size, angle=90),
panel.spacing = unit(c(0.4,0.4,0.4,0.4),"cm"),
plot.margin = unit(c(0.5,0.5,0.5,0.5),"cm"),
legend.position="none",
plot.title = element_text(lineheight=.8, hjust=0.5, size= font_size))

plot2<-ggplot()+
geom_point(data=efdr, aes(x=e.pvalue.DESEQ2 , y=raw.pvalue.1),color="black", size=2,shape=16)+
geom_line(data=efdr, aes(x=e.pvalue.DESEQ2 , y=raw.pvalue.1),color="black", size=0.1,linetype=3)+
scale_x_continuous(name="empirical FDR", limits = c(0, 0.005), breaks=seq(0, 0.05, 0.0005))+
scale_y_continuous(name="nominal P value", limits = c(0, 0.002), breaks=seq(0, 0.002, 0.0005))+
ggtitle("Differential gene expression eFDR \n DeSeq2 WALD test")+
theme_bw()+
theme(panel.grid= element_blank(),
panel.background = element_blank(),
panel.grid.minor = element_blank(),
panel.grid.major = element_line(color="lightgray"),
plot.background = element_blank(),
axis.text.y=element_text(color="black", size=font_size),
axis.text.x=element_text(color="black", size=font_size, angle=90),
panel.spacing = unit(c(0.4,0.4,0.4,0.4),"cm"),
plot.margin = unit(c(0.5,0.5,0.5,0.5),"cm"),
legend.position="none",
plot.title = element_text(lineheight=.8, hjust=0.5, size= font_size))

plot3<-ggplot()+
geom_point(data=efdr, aes(x=e.pvalue.DESEQ2.1 , y=raw.pvalue.1),color="black", size=2,shape=16)+
geom_line(data=efdr, aes(x=e.pvalue.DESEQ2.1 , y=raw.pvalue.1),color="black", size=0.1,linetype=3)+
scale_x_continuous(name="empirical FDR", limits = c(0, 0.005), breaks=seq(0, 0.05, 0.0005))+
scale_y_continuous(name="nominal P value", limits = c(0, 0.002), breaks=seq(0, 0.002, 0.0005))+
ggtitle("Differential gene expression eFDR \n LTR test")+
theme_bw()+
theme(panel.grid= element_blank(),
panel.background = element_blank(),
panel.grid.minor = element_blank(),
panel.grid.major = element_line(color="lightgray"),
plot.background = element_blank(),
axis.text.y=element_text(color="black", size=font_size),
axis.text.x=element_text(color="black", size=font_size, angle=90),
panel.spacing = unit(c(0.4,0.4,0.4,0.4),"cm"),
plot.margin = unit(c(0.5,0.5,0.5,0.5),"cm"),
legend.position="none",
plot.title = element_text(lineheight=.8, hjust=0.5, size= font_size))


cowplot::plot_grid(plot1, plot2, plot3, nrow=1)
```

## sessionInfo

```
sessionInfo()
```

```
## R version 4.3.1 (2023-06-16)
## Platform: x86_64-pc-linux-gnu (64-bit)
## Running under: Ubuntu 20.04.6 LTS
## 
## Matrix products: default
## BLAS:   /usr/lib/x86_64-linux-gnu/blas/libblas.so.3.9.0 
## LAPACK: /usr/lib/x86_64-linux-gnu/lapack/liblapack.so.3.9.0
## 
## locale:
##  [1] LC_CTYPE=en_US.UTF-8       LC_NUMERIC=C              
##  [3] LC_TIME=en_US.UTF-8        LC_COLLATE=en_US.UTF-8    
##  [5] LC_MONETARY=en_US.UTF-8    LC_MESSAGES=en_US.UTF-8   
##  [7] LC_PAPER=en_US.UTF-8       LC_NAME=C                 
##  [9] LC_ADDRESS=C               LC_TELEPHONE=C            
## [11] LC_MEASUREMENT=en_US.UTF-8 LC_IDENTIFICATION=C       
## 
## time zone: America/New_York
## tzcode source: system (glibc)
## 
## attached base packages:
##  [1] parallel  grid      stats4    stats     graphics  grDevices utils    
##  [8] datasets  methods   base     
## 
## other attached packages:
##  [1] MOFA2_1.10.0                MultiAssayExperiment_1.26.0
##  [3] emmeans_1.8.6               lme4_1.1-33                
##  [5] Matrix_1.5-4.1              ggmanh_1.4.0               
##  [7] doParallel_1.0.17           iterators_1.0.14           
##  [9] foreach_1.5.2               bigmemory_4.6.1            
## [11] gridExtra_2.3               kableExtra_1.3.4           
## [13] ggsignif_0.6.4              data.table_1.14.8          
## [15] goseq_1.52.0                geneLenDataBase_1.36.0     
## [17] BiasedUrn_2.0.10            car_3.1-2                  
## [19] carData_3.0-5               ggpubr_0.6.0               
## [21] reshape2_1.4.4              htmlwidgets_1.6.2          
## [23] lubridate_1.9.2             forcats_1.0.0              
## [25] stringr_1.5.0               purrr_1.0.1                
## [27] readr_2.1.4                 tidyr_1.3.0                
## [29] tibble_3.2.1                tidyverse_2.0.0            
## [31] plotly_4.10.2               flashClust_1.01-2          
## [33] ComplexHeatmap_2.16.0       VennDiagram_1.7.3          
## [35] futile.logger_1.4.3         readxl_1.4.2               
## [37] DESeq2_1.40.1               SummarizedExperiment_1.30.2
## [39] Biobase_2.60.0              MatrixGenerics_1.12.1      
## [41] matrixStats_1.0.0           GenomicRanges_1.52.0       
## [43] GenomeInfoDb_1.36.0         IRanges_2.34.0             
## [45] S4Vectors_0.38.1            BiocGenerics_0.46.0        
## [47] GGally_2.1.2                cowplot_1.1.1              
## [49] edgeR_3.42.4                limma_3.56.2               
## [51] ggplot2_3.4.2               dplyr_1.1.2                
## 
## loaded via a namespace (and not attached):
##   [1] bitops_1.0-7             httr_1.4.6               webshot_0.5.4           
##   [4] RColorBrewer_1.1-3       tools_4.3.1              backports_1.4.1         
##   [7] utf8_1.2.3               R6_2.5.1                 HDF5Array_1.28.1        
##  [10] lazyeval_0.2.2           uwot_0.1.14              mgcv_1.8-42             
##  [13] rhdf5filters_1.12.1      GetoptLong_1.0.5         withr_2.5.0             
##  [16] prettyunits_1.1.1        cli_3.6.1                formatR_1.14            
##  [19] sandwich_3.0-2           labeling_0.4.2           sass_0.4.6              
##  [22] mvtnorm_1.2-2            askpass_1.1              Rsamtools_2.16.0        
##  [25] systemfonts_1.0.4        svglite_2.1.1            rstudioapi_0.14         
##  [28] RSQLite_2.3.1            generics_0.1.3           shape_1.4.6             
##  [31] BiocIO_1.10.0            GO.db_3.17.0             fansi_1.0.4             
##  [34] abind_1.4-5              lifecycle_1.0.3          multcomp_1.4-23         
##  [37] yaml_2.3.7               rhdf5_2.44.0             BiocFileCache_2.8.0     
##  [40] Rtsne_0.16               blob_1.2.4               crayon_1.5.2            
##  [43] dir.expiry_1.8.0         lattice_0.21-8           GenomicFeatures_1.52.0  
##  [46] KEGGREST_1.40.0          magick_2.7.4             pillar_1.9.0            
##  [49] knitr_1.43               rjson_0.2.21             boot_1.3-28.1           
##  [52] estimability_1.4.1       codetools_0.2-19         glue_1.6.2              
##  [55] pdftools_3.3.3           qpdf_1.3.2               vctrs_0.6.2             
##  [58] png_0.1-8                cellranger_1.1.0         gtable_0.3.3            
##  [61] cachem_1.0.8             xfun_0.39                S4Arrays_1.0.4          
##  [64] mime_0.12                coda_0.19-4              survival_3.5-5          
##  [67] pheatmap_1.0.12          statmod_1.5.0            TH.data_1.1-2           
##  [70] nlme_3.1-162             pbkrtest_0.5.2           bit64_4.0.5             
##  [73] progress_1.2.2           filelock_1.0.2           bslib_0.5.0             
##  [76] colorspace_2.1-0         DBI_1.1.3                tidyselect_1.2.0        
##  [79] bit_4.0.5                compiler_4.3.1           curl_5.0.1              
##  [82] rvest_1.0.3              basilisk.utils_1.12.1    xml2_1.3.4              
##  [85] DelayedArray_0.26.3      rtracklayer_1.60.0       scales_1.2.1            
##  [88] rappdirs_0.3.3           digest_0.6.31            minqa_1.2.5             
##  [91] rmarkdown_2.22           basilisk_1.12.0          XVector_0.40.0          
##  [94] htmltools_0.5.5          pkgconfig_2.0.3          highr_0.10              
##  [97] dbplyr_2.3.2             fastmap_1.1.1            rlang_1.1.1             
## [100] GlobalOptions_0.1.2      farver_2.1.1             jquerylib_0.1.4         
## [103] zoo_1.8-12               jsonlite_1.8.5           BiocParallel_1.34.2     
## [106] RCurl_1.98-1.12          magrittr_2.0.3           GenomeInfoDbData_1.2.10 
## [109] Rhdf5lib_1.22.0          munsell_0.5.0            Rcpp_1.0.10             
## [112] reticulate_1.29          stringi_1.7.12           zlibbioc_1.46.0         
## [115] MASS_7.3-60              plyr_1.8.8               ggrepel_0.9.3           
## [118] bigmemory.sri_0.1.6      Biostrings_2.68.1        splines_4.3.1           
## [121] hms_1.1.3                circlize_0.4.15          locfit_1.5-9.7          
## [124] uuid_1.1-0               biomaRt_2.56.0           futile.options_1.0.1    
## [127] XML_3.99-0.14            evaluate_0.21            lambda.r_1.2.4          
## [130] nloptr_2.0.3             tzdb_0.4.0               reshape_0.8.9           
## [133] clue_0.3-64              broom_1.0.4              xtable_1.8-4            
## [136] restfulr_0.0.15          rstatix_0.7.2            viridisLite_0.4.2       
## [139] memoise_2.0.1            AnnotationDbi_1.62.1     GenomicAlignments_1.36.0
## [142] cluster_2.1.4            corrplot_0.92            timechange_0.2.0
```
